# Addressing tobacco industry influence in tobacco-growing countries

**DOI:** 10.2471/BLT.23.290219

**Published:** 2023-10-31

**Authors:** Raphael A Lencucha, Nuntavarn Vichit-Vadakan, Roengrudee Patanavanich, Rob Ralston

**Affiliations:** a School of Physical and Occupational Therapy, McGill University, 3630 Promenade Sir William Osler, Montreal, QC H3G 1Y5, Canada.; b School of Global Studies, Thammasat University, Pratumtani, Thailand.; c Faculty of Medicine, Mahidol University, Bangkok, Thailand.; d School of Social and Political Science, University of Edinburgh, Edinburgh, Scotland.

## Abstract

Protecting policy-making from tobacco industry influence is central to effective tobacco control governance. The inclusion of industry actors as stakeholders in policy processes remains a crucial avenue to corporate influence. This influence is reinforced by the idea that the tobacco industry is a legitimate partner to government in regulatory governance. Addressing the influence of the tobacco industry demands a focus on the government institutions that formalize relationships between industry and policy-makers. Industry involvement in government institutions is particularly relevant in tobacco-growing countries, where sectors of government actively support tobacco as an economic commodity. In this paper, we discuss how controlling tobacco industry influence requires unique consideration in tobacco-growing countries. In these countries, there is a diverse array of companies that support tobacco production, including suppliers of seeds, equipment and chemicals, as well as transportation, leaf buying and processing, and manufacturing companies. The range of companies that operate in these contexts is particular and so is their engagement within political institutions. For governments wanting to support alternatives to tobacco growing (Article 17 of the Framework Convention for Tobacco Control), we illustrate how implementing Article 5.3, aimed at protecting tobacco control policies from tobacco industry interference, is fundamental in these countries. Integrating Article 5.3 with Article 17 will (i) strengthen policy coherence, ensuring that alternative livelihood policies are not undermined by tobacco industry interference; (ii) foster cross-sector collaboration addressing both tobacco industry interference and livelihood development; and (iii) enhance accountability and transparency in tobacco control efforts.

## Résumé

Éviter que l'élaboration de politiques soit influencée par l'industrie du tabac est essentiel pour assurer une gestion efficace de la lutte antitabac. L'inclusion d’acteurs de l'industrie comme parties prenantes des processus législatifs demeure l'un des principaux leviers employés par les sociétés. Cette influence est renforcée par l'idée que l'industrie du tabac constitue un partenaire légitime du gouvernement dans le domaine de la gouvernance réglementaire. Pour lutter contre l'ingérence du secteur, il faut se concentrer sur les institutions gouvernementales qui officialisent les relations entre l'industrie et les responsables politiques. L'implication de l'industrie dans les institutions publiques est surtout observée dans les pays producteurs de tabac, où certains niveaux de gouvernement soutiennent activement le tabac en tant que bien économique. Dans le présent document, nous abordons le fait qu'une lutte contre l'influence de l'industrie du tabac requiert une attention particulière dans les pays producteurs, où un large éventail d'entreprises encouragent la production de tabac: approvisionnement en semences, équipements et produits chimiques, mais aussi transport, achat des feuilles, transformation et fabrication. La variété des entreprises œuvrant dans de tels contextes est spécifique, tout comme leur engagement au sein des organes politiques. Pour les gouvernements qui souhaitent privilégier les alternatives à la production de tabac (selon l'article 17 de la Convention-cadre pour la lutte antitabac), nous montrons à quel point l'application de l'article 5.3, qui vise à protéger les politiques de lutte antitabac contre l'ingérence de l'industrie, est fondamentale dans ces pays. Intégrer l'article 5.3 dans l'article 17 permettra (i) de renforcer la cohérence des politiques en vigueur, afin que les politiques relatives à des activités de remplacement ne soient pas contrecarrées par l'industrie du tabac; (ii) de favoriser la collaboration entre les secteurs pour lutter contre l'influence de l'industrie tout en développant des moyens de subsistance; et enfin, (iii) d'accroître la responsabilisation et la transparence dans les efforts menés dans la lutte antitabac.

## Resumen

Proteger la formulación de políticas contra la influencia de la industria tabacalera es fundamental para una gobernanza eficaz del control del tabaco. La inclusión de los actores de la industria como partes interesadas en los procesos políticos sigue siendo una vía crucial para la influencia corporativa. Esta influencia se ve reforzada por la idea de que la industria tabacalera es un asociado legítimo del gobierno en la gobernanza reglamentaria. La lucha contra la influencia de la industria tabacalera exige centrarse en las instituciones gubernamentales que formalizan las relaciones entre la industria y los responsables de formular políticas. La participación de la industria en las instituciones gubernamentales es especialmente relevante en los países productores de tabaco, donde algunos sectores del gobierno apoyan activamente el tabaco como un bien económico. En este documento, se analiza cómo el control de la influencia de la industria tabacalera requiere una consideración especial en los países productores de tabaco. En estos países, existe una gran variedad de empresas que apoyan la producción de tabaco, incluidos los proveedores de semillas, equipos y productos químicos, así como las empresas de transporte, de compra de hojas y de procesamiento y fabricación. La variedad de empresas que operan en estos contextos es particular y también lo es su compromiso dentro de las instituciones políticas. Para los gobiernos que desean apoyar alternativas al cultivo de tabaco (es decir, el artículo 17 del Convenio Marco para el Control del Tabaco), se ilustra cómo la aplicación del artículo 5.3, destinado a proteger las políticas de control del tabaco de la interferencia del tabaco, es fundamental en estos países. La integración del artículo 5.3 con el artículo 17 (i) reforzará la coherencia política, asegurando que las políticas de medios de vida alternativos no se vean perjudicadas por la interferencia de la industria tabacalera; (ii) fomentará la colaboración intersectorial al abordar tanto la interferencia de la industria tabacalera como el desarrollo de los medios de vida; y (iii) mejorará la rendición de cuentas y la transparencia en los esfuerzos de control del tabaco.

## ملخص

إن حماية عملية وضع السياسات من تأثير صناعة التبغ هو أمر أساسي للإدارة الفعالة للسيطرة على التبغ. إن إدراج الجهات الفاعلة في الصناعة كأصحاب للمصلحة في عمليات السياسة، يظل وسيلة حاسمة لتأثير الشركات. ويتعزز هذا التأثير من خلال فكرة أن صناعة التبغ هي شريك شرعي للحكومة في الإدارة التنظيمية. تتطلب معالجة تأثير صناعة التبغ التركيز على المؤسسات الحكومية التي تضفي الطابع الرسمي على العلاقات بين الصناعة وواضعي السياسات. إن مشاركة الصناعة في المؤسسات الحكومية لها أهمية خاصة في الدول التي تزرع التبغ، حيث تدعم القطاعات الحكومية بنشاط التبغ كسلعة اقتصادية. نحن نناقش في هذه الورقة كيف أن السيطرة على تأثير صناعة التبغ تتطلب اهتمامًا فريدًا في الدول التي تزرع التبغ. في هذه الدول، هناك مجموعة متنوعة من الشركات التي تدعم إنتاج التبغ، بما في ذلك موردي البذور، والمعدات، والمواد الكيميائية، فضلا عن شركات النقل، وشراء الأوراق وتجهيزها وتصنيعها. إن نطاق الشركات التي تعمل في هذه الأوضاع هو نطاق محدد، وكذلك مشاركتها في المؤسسات السياسية. بالنسبة للحكومات الراغبة في دعم بدائل لزراعة التبغ (أي المادة 17 من الاتفاقية الإطارية لمكافحة التبغ)، نوضح كيف أن تنفيذ المادة 5.3، التي تهدف إلى حماية سياسات مكافحة التبغ من تدخل التبغ، هو أمر حيوي في هذه الدول. سيؤدي دمج المادة 5.3 مع المادة 17 إلى (1) تعزيز تماسك السياسات، وضمان عدم تقويض سياسات سبل العيش البديلة بسبب تدخل صناعة التبغ؛ و(2) تعزيز التعاون بين القطاعات لمعالجة تدخل صناعة التبغ وتنمية سبل العيش؛ و(3) تعزيز المساءلة والشفافية في جهود مكافحة التبغ.

## 摘要

保护政策制定免受烟草行业影响是进行有效烟草控制治理的核心。在政策制定过程将行业参与者作为利益攸关方考虑，仍然是发挥企业影响力的重要途径。烟草行业是政府在监管治理方面的合法合作伙伴，这一观念加强了这种影响。要解决烟草行业的影响的问题，就需要把重心放在使烟草行业与政策制定者之间关系正规化的政府机构上。在烟草种植国，烟草行业参与到政府机构的相关政策制定过程尤其有意义，因为这些国家的政府部门积极支持烟草作为一种经济作物。在本文中，我们讨论了如何控制烟草行业的影响，以及这需要烟草种植国考虑哪些特殊考虑因素。在这些国家，支持烟草生产的公司种类繁多，包括种子、设备和化学品供应商，以及运输、烟叶购买和加工制造公司。在这些背景下运营的公司的业务范围是特殊的，它们在政治机构内的参与也是如此。对于愿意支持烟草替代农作物种植（即《烟草控制框架公约》第 17 条）的政府，我们说明了实施第 5.3 条准则在这些国家的重要性，该准则旨在保护烟草控制政策免受烟草行业的干扰。将第 5.3 条与第 17 条结合起来，将 (i) 加强政策的一致性，确保替代民生政策不受烟草行业干扰的影响；(ii) 促进跨部门合作，解决烟草行业干扰和生计发展问题；以及 (iii) 加强烟草控制工作的问责制并提高其透明度。

## Резюме

Защита процесса принятия стратегических решений от влияния табачной промышленности является центральным элементом эффективного управления борьбой с потреблением табака. Включение представителей промышленности в качестве заинтересованных сторон в процессы формирования политики остается одним из важнейших направлений корпоративного влияния. Это влияние подкрепляется идеей о том, что табачная промышленность является легитимным партнером правительства в сфере регулирования. Для решения проблемы влияния табачной промышленности необходимо обратить внимание на государственные структуры, которые формализуют отношения между промышленностью и директивными органами. Участие промышленности в работе государственных структур особенно актуально в странах, выращивающих табак, где государственные структуры активно поддерживают табак как экономический товар. В данной статье рассматривается вопрос о том, как контроль над влиянием табачной промышленности требует особого подхода в странах, выращивающих табак. В этих странах существует целый ряд компаний, поддерживающих производство табака, включая поставщиков семян, оборудования и химикатов, а также транспортные компании, компании, занимающиеся закупкой и переработкой листьев, и производственные компании. Спектр работающих в этих условиях компаний специфичен, как и их взаимодействие с политическими структурами. Для государств, желающих поддержать альтернативы выращиванию табака (это статья 17 Рамочной конвенции по борьбе против табака), показано, как выполнение статьи 5.3, направленной на защиту политики борьбы против табака от вмешательства табачных компаний, имеет принципиальное значение в этих странах. Интеграция статьи 5.3 со статьей 17 (i) повысит согласованность политики, что позволит не подвергать политику альтернативных источников средств к существенному вмешательству табачной промышленности; (ii) будет способствовать межсекторному сотрудничеству, направленному как на вмешательство табачной промышленности, так и на развитие источников средств к существованию; (iii) повысит подотчетность и прозрачность усилий по борьбе с потреблением табака.

## Introduction

In 2023, the World Health Organization’s (WHO) campaign World No Tobacco Day focuses on the importance of finding alternatives to tobacco growing.^1^ The campaign “calls on governments … to step up legislation, develop policies and strategies, and enable market conditions for farmers to shift to food crops that would provide them … with a better life.” This campaign stems from Article 17 of WHO’s Framework Convention for Tobacco Control (FCTC) which requires signatories to support alternatives to tobacco growing.

Tobacco growing is largely detrimental to ecosystems, the livelihoods of farming households, and takes up valuable agricultural land that could be used for food-based crops.^2^ Despite these known harms, tobacco remains a prominent economic commodity for many governments. The difficulty in promoting alternatives is, in part, due to the tobacco industry's influence on government policy.^3^^–^^5^

Research on the commercial determinants of health illustrates that industry employs predictable strategies to protect their core business against government regulation.^6^ Common strategies include lobbying government officials and efforts to shape public perception through advertising, sponsorship and promotion. These strategies have influenced the public's perception of the relationship between government and market, often reinforcing the belief that less government regulation and more market freedom are beneficial for society.^7^ Another way that industry exerts influence is by integrating themselves into government institutions, aiming to shape public policy and programmes from within.^8^ These strategies are commonly used in countries where tobacco is cultivated, wherein the interests of the industry and government policies and programmes reinforce each other.

The tobacco industry has systematically promoted and leveraged the claim that tobacco contributes positively to economies. They use this claim to oppose tobacco control and position themselves as integral collaborators in government development strategies. This effort is exemplified by the support that industry provides to the International Tobacco Growers Association, an association advancing the narrative that tobacco control harms the livelihoods of tobacco farmers.^9^^,^^10^ As a result of these efforts, many governments view tobacco farming as a path to economic growth and improvement in rural livelihoods.^11^

An important entry point for industry influence is formal inclusion of the tobacco industry in institutions and government agencies. Through such inclusion, norms that support industry-government interactions become institutionalized. Such norms include the view that the industry contributes to economic development, employment, and is an important partner in government action.^8^^,^^12^ This institutionalization of norms serves to sustain and legitimize industry influence over government action. Efforts to protect government from industry influence, particularly health-harming industries like tobacco, requires a focus on industry involvement with government institutions. We know that industry seeks legitimacy in these spaces, because when governments perceive the industry as a legitimate stakeholder, the industry is able to influence policy agendas.

This integration of the industry within government institutions is particularly relevant in tobacco-growing countries. The pursuit of alternatives to tobacco cultivation, and more broadly, comprehensive tobacco control across sectors is hampered by the tobacco industry interests. In this paper, we aim to illustrate that controlling tobacco industry influence requires unique consideration in tobacco-growing countries. Subsequently, building on previous research, we argue that the effective implementation of Article 5.3 of the FCTC, that is, protecting tobacco control policies from tobacco industry interference in tobacco-growing countries, requires consideration of this unique context. We argue that Article 5.3 is foundational to the pursuit of Article 17 of the FCTC. Simultaneously, the implementation of Article 5.3 must be tailored to accommodate the intricacies of industry-government relationships within the distinct institutional contexts of these countries.

## Tobacco-growing countries 

### Tobacco industry

In tobacco-growing countries, what constitutes the tobacco industry is unique. There exists a diverse array of companies supporting tobacco production. Therefore, these countries require unique considerations when examining and addressing the influence of the tobacco industry. The deep integration of tobacco industry interests along the supply chain serves as an important driver of pro-tobacco agendas.^3^^,^^13^

While cigarette companies drive the supply of tobacco leaf, the supply chain itself has several levels of companies that facilitate this supply. In addition to cigarette companies like British American Tobacco or Philip Morris International, there are companies that supply tobacco growers with essentials, including barn materials, tools and equipment, tobacco seeds, fertilizers, chemicals and packaging materials. There are also transportation companies and other services that support tobacco supply. Each of these suppliers has a vested interest in tobacco as an economic commodity. Often, these companies are interconnected along the supply chain through leaf-buying companies. Generally, between 70%–90% of smallholder tobacco farmers grow tobacco through contracts with leaf-buying companies.^14^^–^^18^ These companies often procure and distribute inputs like seeds, fertilizer, pesticides and herbicides, and manage the transportation of leaf to market.^19^^,^^20^

### Tobacco governance

Various ministries and agencies oversee the tobacco supply chain; however, their collective objective often leans towards maintaining tobacco as an economic commodity.^3^^,^^21^^,^^22^ A notable component of this institutional oversight is the presence of tobacco boards. These boards have diverse membership, including ministries of agriculture, commerce, finance, and industry as well as farmer associations.^23^ The boards are responsible for overseeing the grading scheme that determines the correlation between quality and price, managing the auction system and handling other aspects related to the sale of tobacco leaf. Importantly, these boards have an explicit mandate to promote, protect and maintain the production, sale and preparation for subsequent use and export of tobacco.^24^ The following statement from the Tobacco Board of Zambia illustrates how these institutions are committed to tobacco, and resist shifts away from tobacco growing:

“Any attempt to stop tobacco production will simply enhance poverty in the rural household[s] that depend on tobacco production for their livelihood.”

Included in the institutional network are other important agencies like government-supported research agencies with the mandate to support the technical aspects of tobacco growing, and commissions that have a mandate to regulate the production and marketing of tobacco.^25^ Importantly, this network of institutions harbours both formal and informal ties with the tobacco industry. For example, in Malawi, a major tobacco-growing country, the Agricultural Research and Extension Trust has two members of the agriculture ministry sitting next to one representative from the tobacco industry and four representatives from the Tobacco Association of Malawi, an association with linkages to the International Tobacco Growers Association.^13^^,^^26^ The interaction between government and industry is explicitly supported by these associations and agencies. For example, when addressing challenges posed by the expanding tobacco control movement, the chief executive officer of the Agricultural Research and Extension Trust mentioned “through continued interaction with various stakeholders we continue to get important feedback which helps us surmount the challenges and achieve our goals.”^27^

An important part of the challenge of preventing tobacco industry influence stems from the efforts of influential transnational tobacco companies to normalize their products and practices.^28^ For example, a leaked 10-year strategic plan from Philip Morris International in 2014 revealed that a key strategy for the company is to “build on existing, and foster future, stakeholder relations with international organizations, politicians … etc. to further expand communications and engagement opportunities.”^29^ Philip Morris International has funded the Foundation for a Smoke-Free World which is active in Malawi, partnering with the government to promote the idea that the tobacco industry can be a constructive player in seeking alternatives to tobacco cultivation. This intricate network of relationships between tobacco industry interests and the government not only sustains tobacco supply but also creates avenues for the industry to oppose tobacco control. The 10-year plan captures this dynamic explicitly, where Philip Morris International has outlined a primary objective: to “proactively increase our stakeholder base to equip us to better shape the future sales environment” and make it “politically unattractive to implement excessive sales regulations/restrictions … via engaging directly with policy-makers, media and third parties.”^29^ Similar initiatives can be observed at the International Labour Organization, where the major tobacco companies partnered to position themselves as champions of the anti-child labour movement. By doing so, they aim to gain favour with governments and attempt to portray themselves as legitimate partners.^30^ In countries, such as China and Thailand, where the government often exercises direct and comprehensive control over tobacco supply, transnational companies still maintain involvement at various stages along the supply chain. Box 1 presents the complex dynamic involved even when the Thai government has direct control over supply.

Box 1 **The tobacco system in Thailand: state control, corporate influence and farmers' predicament**

In Thailand, the tobacco system consists of four major entities: the finance ministry, Tobacco Authority of Thailand, tobacco farmers and multinational tobacco companies. By law, the finance ministry governs the entire tobacco system, which includes the licensing and registration for tobacco growing, leaf purchasing, tobacco manufacturing, and the sale, import and export of tobacco products.^31^ Tobacco Authority of Thailand, a state-owned company under the finance ministry, is the only legal entity authorized to produce cigarettes in the country. Furthermore, Tobacco Authority of Thailand has developed a tobacco contract farming system and holds the authority to assign tobacco quotas to local farmers.^32^ While private tobacco companies are not allowed to grow or manufacture tobacco in Thailand, they engage in the process by collaborating with local curing firms and establishing their own contract farming system for leaf purchasing and export.

Multinational tobacco companies have a long-standing relationship with Thai tobacco farmers. The 1990–1992 action plan from Philip Morris explicitly aimed to identify Thai farmers’ groups and promote their membership in the International Tobacco Growers’ Association, a tobacco industry front group.^33^ Thailand Tobacco Growers' Association is currently a member of International Tobacco Growers’ Association. Moreover, Philip Morris International has donated to Thai tobacco farmers through various projects. In 2022, Philip Morris International funded to multiple tobacco farming associations for projects such as scholarship programs for the children of tobacco growers, and capacity-building training sessions for female tobacco farmers.^34^ Many of these tobacco farmer groups have opposed certain tobacco control measures, like tobacco taxes and ingredient regulations, citing economic repercussions.^35^

Notwithstanding the robust ties between the Tobacco Farmers’ Association and the multinational tobacco industry, nearly 60% of local Thai tobacco farmers have expressed a desire to cease tobacco cultivation. This sentiment stems from factors such as reduced tobacco cultivation quotas from the Tobacco Authority of Thailand, health and quality-of-life concerns, the effects of natural disasters, and a shift in the younger generation's reluctance to pursue careers in tobacco farming.^36^

These examples illustrate the intricate interweaving of government-industry relationships within the institutional frameworks in tobacco-growing countries. The examples further underscore that the dynamic isn't merely about the connections between government and industry, but also about the mandates that both validate and reinforce these relationships.^37^ These institutional dynamics influence the ability of health ministries to garner support from other sectors when implementing the multisectoral provisions of the FCTC. Considering tobacco industry interests as stakeholders in government decision-making enables commercial entities to influence governmental actions. In many tobacco-growing countries, FCTC-compliant tobacco legislation has largely stalled due to the influence of economic arguments and interests. The tireless efforts by champions within government and tobacco control advocates have faced resistance from the powerful economic sector, which consistently contends that tobacco plays a crucial role in the country’s economic development.^21^^,^^38^

### Implementing Article 17

To safeguard the health and livelihoods of tobacco farmers, workers and their communities, Article 17 of the FCTC requires Parties to promote economically viable alternatives:

“Parties shall, in cooperation with each other and with competent international and regional intergovernmental organizations, promote, as appropriate, economically viable alternatives for tobacco workers, growers and, as the case may be, individual sellers.”

Implementing Article 17 aligns with several sustainable development goals (SDGs), including SDG 1 (reducing poverty), SDG 3 (improving health and well-being), SDG 5 (promoting gender equality), SDG 8 (ensuring decent work and employment), SDG 12 (encouraging responsible consumption and production) and SDG 15 (protecting life on land).^39^ Many governments have initiated programmes that support alternatives to tobacco growing. For example, in 2017, the Brazilian National Programme of Diversification in Tobacco-Growing Areas supported 11 000 families in six tobacco-growing states to diversify their livelihoods, and protect the health of tobacco farmers and natural resources in their communities. In 2000, Malaysia introduced kenaf as an alternative to tobacco. The Malaysian government provided financial incentives for inputs and mechanization support to kenaf growers, which increased the kenaf cultivation area to 1364 hectares, with a total of 928 kenaf growers, in 2020.^40^ Despite these efforts by countries where tobacco is grown, Article 17 remains one of the least-implemented obligations of the WHO FCTC.^40^

### Implementing Article 5.3

The FCTC is distinctive among framework conventions in its recognition of the potential for an industry to undermine its objectives. Article 5.3 is a policy instrument within the FCTC, designed to protect policy-making from commercial and other vested interests of the tobacco industry.^41^ Within the general obligations of Article 5.3, the guidelines call on all departments within government to be accountable to the principles that protect tobacco control from industry influence. For instance, the guidelines state that “Any government branch (executive, legislative and judiciary) responsible for setting and implementing tobacco control policies and for protecting those policies against tobacco industry interests should be accountable.”^42^

Article 5.3, as a policy tool, comprises both procedural and substantive guidelines aimed at establishing and maintaining a specific model of regulatory governance. Procedural guidelines are those designed to shape the internal workings of government and how the government interacts with non-state actors. These guidelines are designed to limit interactions between public officials and the tobacco industry, ensuring any necessary engagements are conducted transparently and with accountability. Substantive guidelines are those used to affect policy outcomes such as regulation or subsidies^43^ and shape the political economy of the tobacco industry, including the removal of tax exemptions and restrictions on state investment in the tobacco industry. These procedural and substantive guidelines are designed to be complementary, with decision-making procedures supporting the pursuit of substantive goals such as reducing the supply of tobacco. 

While Article 5.3 has been framed as the backbone of the FCTC,^44^ its relevance to the implementation of other articles within the treaty remains underexplored, especially for Article 17. Articles 5.3 and 17 are related in their focus on the political economy of tobacco production. As noted, the guidelines for the implementation of Article 5.3 require governments to avoid preferential treatment and introduce mechanisms to remove incentives to the tobacco industry.^42^ In relation to Article 17, these rules and tools apply to all actors along the tobacco supply chain, such as the trade associations and leaf buyers.

The status of Article 5.3 as a general obligation of the FCTC means that it applies to all sectors of government, with political institutions across trade, finance and agriculture subject to its norms, rules and procedures. The article contains procedures that are designed to shape how government, as a whole, interacts with the tobacco industry and also frames the use of other instruments. In theory, Article 5.3 should shape the use of other procedural instruments, such as stakeholder consultation, commissions and advisory committees, according to its normative goal of protecting policy-making from tobacco industry interests. Realizing the transformational potential of Article 5.3 has proved highly challenging.^45^ This challenge stems not only from the need to shift from established governance mechanisms and practices, but also from the deeply rooted institutional structures granting the tobacco industry formal access to government.^8^

To implement Article 5.3 in tobacco-growing contexts requires understanding the sites of connection between industry and government, as illustrated above. One starting point is the establishment of multisectoral coordinating mechanisms as required by Article 5.2 of the Convention. These coordinating mechanisms have important potential not only to shape the operations of all sectors of government in the implementation of FCTC provisions, but also to shift the norms of industry-government relations. Often these mechanisms illustrate existing tensions in the governance norms held by different sectors. In the Philippines, the Interagency Committee–Tobacco was created as part of the Tobacco Regulation Act of 2003 and is the sole body charged with implementing the Act.^12^ The Department of Trade and Industry, whose mandate has involved support for the tobacco industry, chairs the Interagency Committee–Tobacco. The committee also includes the National Tobacco Administration, which is mandated by law to promote the development of the tobacco industry on the agriculture side. Additionally, the Philippine Tobacco Institute, which represents the tobacco industry, is a part of thecommittee. The Philippine Tobacco Institute has filed suits against the Department of Health for their tobacco control efforts, leading civil society organizations to pursue a campaign to remove them from the Interagency Committee–Tobacco.^46^ The situation with the National Commission for FCTC Implementation in Brazil presents a nuanced parallel. While the health sector leads the commission, the inclusion of the ministry of agribusiness in the thirteen-ministry coordinating mechanism poses challenges to tobacco control efforts. Participants in a study on this national commission noted that the ministry of agribusiness worked closely with tobacco companies, with a mandate to support these companies.^47^ These examples illustrate, the potential of Article 5.3 to reorient industry-government relations towards supply and demand reduction. They also highlight the challenges that arise when government sectors view tobacco as an economic commodity.

## Conclusion

Addressing the interactions between the tobacco industry and governments in tobacco-growing countries presents unique challenges. However, addressing these challenges is essential if governments are to effectively implement Article 17 and other provisions of the FCTC. To pursue Article 5.3, Parties need to understand the unique institutional landscape within tobacco-growing countries, especially where the industry interacts with government. Many industry actors that support tobacco production and who sit next to government representatives on agencies are not directly tobacco companies. The range of companies with a stake in tobacco growing is broad, and many of the companies interacting with government in relation to tobacco supply are generic agricultural suppliers that could contribute to the pursuit of alternatives to tobacco growing. The 2021 report, *A multi-billion dollar opportunity: repurposing agricultural support to transform food systems*, published by the Food and Agriculture Organization, the United Nations Development Programme and the United Nations Environment Programme, outlines how such agencies and other entities along the agricultural supply chain can be repurposed to pursue food systems transformation.^48^ The recommendations stem from problems associated with current food systems, including the erosion of environments and the concentration of economic benefit with large companies, leaving growers with meagre earnings or debt. These same conditions exist along the tobacco supply chain where farmers earn little to nothing, while cigarette companies generate massive profits.^49^^,^^50^ The 2021 report^48^ highlights that, even amid the complexities inherent in agribusiness systems, there exists a significant opportunity to leverage existing institutions and infrastructure to advocate for food systems that are more equitable, environmentally sustainable and community-focused.

Implementing Article 5.3 in tobacco-growing countries has important implications for controlling industry influence and transforming the political economy of tobacco supply. Mapping the industry's location and understanding its characteristics along the supply chain will be important for identifying which agricultural suppliers are not strictly tobacco companies, but currently have a stake in maintaining support for tobacco production. To shift such companies to other crops requires a shift in institutional mandates away from supporting tobacco growing. Mandates need to support alternatives to tobacco, and work with companies that currently aid agricultural production to support the pursuit of Article 17. These efforts to shift mandates and support alternatives to tobacco requires that leaf-buying companies, as well as those that process and manufacture tobacco, be excluded from governments' agenda-setting efforts from the outset. The fact that the tobacco industry is represented alongside government sectors serves to maintain the emphasis on tobacco as an important economic commodity.^51^

In conclusion, Parties to the FCTC need to disengage from the tobacco industry, including leaf-buying companies and other companies along the supply chain. By doing so, Parties should consider integrating Article 5.3 with Article 17 of the FCTC in the formulation of policies related to economically sustainable alternative livelihoods for tobacco farmers. The integration of Article 5.3 with Article 17 will (i) strengthen policy coherence that ensures alternative livelihood policies are not undermined by tobacco industry interference; (ii) support cross-sector collaboration, addressing both tobacco industry interference and the development of livelihoods; and (iii) enhance accountability and transparency in tobacco control efforts.

## References

[1] World No Tobacco Day 2023: Grow food, not tobacco [internet]. Copenhagen: World Health Organization Regional Office for Europe; 2023. Available from: https://www.who.int/europe/news-room/events/item/2023/05/31/default-calendar/world-no-tobacco-day-2023--we-need-food--not-tobacco [cited 2023 Oct 27].

[2] Lencucha R, Drope J, Magati P, Sahadewo GA. Tobacco farming: overcoming an understated impediment to comprehensive tobacco control. Tob Control. 2022 Mar;31(2):308–12. 10.1136/tobaccocontrol-2021-056564 35241604

[3] Smith J, Lee K. From colonization to globalization: a history of state capture by the tobacco industry in Malawi. Rev Afr Polit Econ. 2018;45(156):186–202. 10.1080/03056244.2018.1431213 31467461 PMC6715304

[4] Otañez MG, Mamudu H, Glantz SA. Global leaf companies control the tobacco market in Malawi. Tob Control. 2007 Aug;16(4):261–9. 10.1136/tc.2006.019273 17652242 PMC2598545

[5] Leppan W, Lecours N, Buckles D. Tobacco control and tobacco farming: separating myth from reality. London: Anthem Press; 2014. 298 pp.

[6] Maani N, van Schalkwyk MCI, Filippidis FT, Knai C, Petticrew M. Manufacturing doubt: assessing the effects of independent vs industry-sponsored messaging about the harms of fossil fuels, smoking, alcohol, and sugar sweetened beverages. SSM Popul Health. 2021 Dec 23;17:101009. 10.1016/j.ssmph.2021.101009 35036514 PMC8749266

[7] Oreskes N, Conway E. The big myth: how American business taught us to loathe government and love the free market. New York: Bloomsbury Publishing; 2023.

[8] Hirpa S, Ralston R, Deressa W, Collin J. ‘They have a right to participate as a stakeholder’: Article 5.3 implementation and government interactions with the tobacco industry in Ethiopia. Tob Control. 2022 Jun;31 Suppl 1:s5–11. 10.1136/tobaccocontrol-2021-056885 35101970 PMC9125371

[9] TobaccoTactics. International Tobacco Growers Association [internet]. Bath: University of Bath; 2020. Available from: https://tobaccotactics.org/article/international-tobacco-growers-association/ [cited 2023 Sep 21].

[10] Assunta M. Tobacco industry’s ITGA fights FCTC implementation in the Uruguay negotiations. Tob Control. 2012 Nov;21(6):563–8. 10.1136/tobaccocontrol-2011-050222 22634569

[11] Tan YL, Dorotheo U. The tobacco control atlas: ASEAN region, fifth edition. Bangkok: Southeast Asia Tobacco Control Alliance (SEATCA); 2021.

[12] Lencucha R, Drope J, Chavez JJ. Whole-of-government approaches to NCDs: the case of the Philippines Interagency Committee—Tobacco. Health Policy Plan. 2015 Sep;30(7):844–52. 10.1093/heapol/czu085 25096748

[13] Otañez MG, Mamudu HM, Glantz SA. Tobacco companies’ use of developing countries’ economic reliance on tobacco to lobby against global tobacco control: the case of Malawi. Am J Public Health. 2009 Oct;99(10):1759–71. 10.2105/AJPH.2008.146217 19696392 PMC2741530

[14] Magati P, Li Q, Drope J, Lencucha R, Labonte R. The economics of tobacco farming in Kenya. Nairobi, Atlanta: Institute of Legislative Affairs and American Cancer Society; 2016.

[15] Makoka D, Drope J, Appau A, Lencucha R. Farm-level economics of tobacco production in Malawi. Lilongwe, Atlanta: Centre for Agricultural Research and Development and American Cancer Society; 2016.

[16] Goma F, Drope J, Zulu R, Li Q, Chelwa G, Labonte R, et al. The economics of tobacco farming in Zambia. Lusaka, Atlanta: University of Zambia and American Cancer Society; 2016.

[17] Drope J, Li Q, Araujo E, Harimurti P, Sahadewo G, Nargis N, et al. The economics of tobacco farming in Indonesia. Washington, DC: World Bank Group; 2017.

[18] Chingosho R, Dare C, van Walbeek C. Tobacco farming and current debt status among smallholder farmers in Manicaland province in Zimbabwe. Tob Control. 2021 Nov;30(6):610–5. 10.1136/tobaccocontrol-2020-055825 32848076 PMC7611881

[19] Scoones I, Mavedzenge B, Murimbarimba F, Sukume C. Tobacco, contract farming, and agrarian change in Zimbabwe. J Agrar Chang. 2018 Jan 1;18(1):22–42. 10.1111/joac.12210

[20] Cipriano IM, Mambo I, Masangano C. Effect of contract tobacco farming on the welfare of smallholder farmers in Angonia District, Mozambique. J Agric Ext Rural Dev. 2017 Dec 31;9(12):292–300.

[21] Smith J, Fang J. ‘If you kill tobacco, you kill Malawi’: structural barriers to tobacco diversification for sustainable development. Sustain Dev. 2020;28(6):1575–83. 10.1002/sd.2106

[22] Lencucha R, Reddy SK, Labonte R, Drope J, Magati P, Goma F, et al. Global tobacco control and economic norms: an analysis of normative commitments in Kenya, Malawi and Zambia. Health Policy Plan. 2018 Apr 1;33(3):420–8. 10.1093/heapol/czy005 29401223 PMC5886138

[23] Labonté R, Lencucha R, Drope J, Packer C, Goma FM, Zulu R. The institutional context of tobacco production in Zambia. Global Health. 2018 Jan 16;14(1):5. 10.1186/s12992-018-0328-y 29338793 PMC5771190

[24] Tobacco board of Zambia [internet]. Lusaka: Tobacco board of Zambia; 2023. Available from: https://www.tbz.co.zm/index.php/about-us [cited 2023 Apr 24].

[25] The tobacco commission [internet]. Lilongwe: The Tobacco Commission; 2023. Available from: https://tc.mw/ [cited 2023 Apr 24].

[26] Gilmore AB, Fooks G, Drope J, Bialous SA, Jackson RR. Exposing and addressing tobacco industry conduct in low-income and middle-income countries. Lancet. 2015 Mar 14;385(9972):1029–43. 10.1016/S0140-6736(15)60312-9 25784350 PMC4382920

[27] Agricultural research and extension trust [Internet]. Lilongwe: Agricultural Research & Extension Trust; 2023. Available from: https://aret.org.mw/ [cited 2023 Feb 10].

[28] Rowell A. Big Tobacco is funding the anti-smoking lobby – but leaked documents reveal the real reason why [internet]. The Conversation. 2018 Mar 18. Available from: http://theconversation.com/big-tobacco-is-funding-the-anti-smoking-lobby-but-leaked-documents-reveal-the-real-reason-why-93087 [cited 2023 Sep 21].

[29] Philip Morris International. 10-year corporate affairs objectives, strategies. 2014. [internet]. News. Philadelphia: DocumentCloud, Contributed by Reuters News. Available from: https://www.documentcloud.org/documents/4333395-10-Year-Corporate-Affairs-Objectives-and [cited 2023 Sep 21].

[30] Tobacco Tactics: International Labour Organization (ILO) [internet]. Bath: University of Bath; 2022. Available from: https://tobaccotactics.org/article/international-labour-organization-ilo/ [cited 2023 Sep 21].

[31] Excise Tax Act, B.E. 2560 (2017). Bangkok: Office of the Council of State; 2017. Available from: http://web.krisdika.go.th/data/document/ext809/809872_0001.pdf [cited 2023 Apr 1].

[32] Promphakping B, Chamaratana T, Somaboot P, Weeranakin P, Promphakping N, Phatchaney K. Why does tobacco agriculture in Thailand persist? Forest and Society. 2021 Oct 11;5(2):543–58. 10.24259/fs.v5i2.13587

[33] Patanavanich R, Glantz SA. How to combat efforts to overturn bans on electronic nicotine delivery systems: lessons from tobacco industry efforts during the 1980s to open closed cigarette markets in Thailand. BMJ Glob Health. 2021 Jan;6(1):e004288. 10.1136/bmjgh-2020-004288 33500264 PMC7843299

[34] 2022 social contributions at a glance [internet]. Stamford: Philip Morris International; 2022. Available from: https://www.pmi.com/resources/docs/default-source/pmi-our-company/2022-social-contributions.pdf?sfvrsn=878cc4b6_2 [cited 2023 Sep 1].

[35] [Tobacco farmer mob stormed the Treasury to oppose extreme tax increase on cigarettes.] Daily News. 2021 Sep 23. Thai. Available from: https://www.dailynews.co.th/news/303530/ [cited 2023 Sep 22].

[36] Phetphum C, Prajongjeep A, Keeratisiroj O, Simsin S, Thawatchaijareonying K. Deteriorating quality of life and a desire to stop growing tobacco among Virginia and Burley tobacco farmers in Thailand. JCO Glob Oncol. 2022 Aug;8(8):e2200180. 10.1200/GO.22.00180 36049151 PMC9470139

[37] Lencucha R, Thow AM. Intersectoral policy on industries that produce unhealthy commodities: governing in a new era of the global economy? BMJ Glob Health. 2020 Aug;5(8):e002246. 10.1136/bmjgh-2019-002246 32816826 PMC7430321

[38] Lencucha R, Drope J, Labonte R. Rhetoric and the law, or the law of rhetoric: how countries oppose novel tobacco control measures at the World Trade Organization. Soc Sci Med. 2016 Sep;164:100–7. 10.1016/j.socscimed.2016.07.026 27475056 PMC4994523

[39] Policy options and recommendations: Articles 17 and 18. Geneva: World Health Organization; 2013. Available from: https://fctc.who.int/publications/m/item/policy-options-and-recommendations-on-economically-sustainable-alternatives-to-tobacco-growing [cited 2023 Oct 27].

[40] 2021 global progress report on implementation of the WHO Framework Convention on Tobacco Control. Geneva: World Health Organization; 2022. Available from: https://fctc.who.int/publications/i/item/9789240041769 [cited 2023 Oct 27].

[41] Ralston R, Hirpa S, Bassi S, Male D, Kumar P, Barry RA, et al. Norms, rules and policy tools: understanding Article 5.3 as an instrument of tobacco control governance. Tob Control. 2022 Jun;31 Suppl 1:s53–60. 10.1136/tobaccocontrol-2021-057159 35393367 PMC9125364

[42] Guidelines for implementation of Article 5.3 of the WHO Framework Convention on Tobacco Control. Geneva: WHO FCTC Convention Secretariat; 2013. Available from: https://www.pmi.com/resources/docs/default-source/pmi-our-company/2022-social-contributions.pdf?sfvrsn=878cc4b6_2 [cited 2023 Oct 27].

[43] Bali AS, Howlett M, Lewis JM, Ramesh M. Procedural policy tools in theory and practice. Policy Soc. 2021 Aug 17;40(3):1–17. 10.1080/14494035.2021.1965379

[44] Assunta M. Global tobacco industry interference index. Bangkok: Global Center for Good Governance in Tobacco Control; 2019. Available from: https://exposetobacco.org/wp-content/uploads/2019/10/GlobalTIIIndex_Report_2019.pdf [cited 2023 Apr 1].

[45] Malone RE, Bialous SA. WHO FCTC article 5.3: promise but little progress. Tob Control. 2014 Jul;23(4):279–80. 10.1136/tobaccocontrol-2014-051817 24928868

[46] Amul GGH, Etter JF. Comparing tobacco and alcohol policies from a health systems perspective: the cases of the Philippines and Singapore. Int J Public Health. 2022 Oct 13;67:1605050. 10.3389/ijph.2022.1605050 36312317 PMC9606809

[47] Lencucha R, Drope J, Bialous SA, Richter AP, Silva VLDCE. [Institutions and the implementation of tobacco control in Brazil]. Cad Saude Publica. 2017 Oct 19;3(33). Portuguese. Suppl 3:e00168315. 10.1590/0102-311X00168315 29069213

[48] Food and Agriculture Organization of the United Nations, United Nations Development Programme, United Nations Environment Programme. A multi-billion dollar opportunity: repurposing agricultural support to transform food systems. Rome: The Food And Agriculture Organization of the United Nations; 2021. 10.4060/cb6683en

[49] Callard C. Follow the money: how the billions of dollars that flow from smokers in poor nations to companies in rich nations greatly exceed funding for global tobacco control and what might be done about it. Tob Control. 2010 Aug;19(4):285–90. 10.1136/tc.2009.035071 20610436 PMC2975973

[50] Chaloupka F, Erika S, Violeta V, Michal S, Maryam M, Drope J, et al. Tobacconomics: cigarette tax scorecard 2020. Chicago: University of Illinois Chicago; 2020.

[51] Fooks GJ. The institutionalization of corporate power within policy. In: Maani N, Pettricrew M, Galea S, editors. The commercial determinants of health. New York: Oxford University Press; 2022. p. 164. 10.1093/oso/9780197578742.003.0017

